# High-frequency ultrasound in anti-aging skin therapy monitoring

**DOI:** 10.1038/s41598-023-45126-y

**Published:** 2023-10-18

**Authors:** Joanna Czajkowska, Jan Juszczyk, Monika Natalia Bugdol, Małgorzata Glenc-Ambroży, Anna Polak, Laura Piejko, Ewa Pietka

**Affiliations:** 1 Faculty of Biomedical Engineering, Silesian University of Technology, 41-800 Zabrze, Poland; 2 Amber Academy Research Center, 44-200 Rybnik, Poland; 3 Jerzy Kukuczka Academy of Physical Education, Institute of Physiotherapy and Health Sciences, 40-065 Katowice, Poland

**Keywords:** Biomedical engineering, Information technology, Diagnosis, Medical imaging, Ultrasonography, Ultrasound

## Abstract

Over the last few decades, high-frequency ultrasound has found multiple applications in various diagnostic fields. The fast development of this imaging technique opens up new diagnostic paths in dermatology, allergology, cosmetology, and aesthetic medicine. In this paper, being the first in this area, we discuss the usability of HFUS in anti-aging skin therapy assessment. The fully automated algorithm combining high-quality image selection and entry echo layer segmentation steps followed by the dermal parameters estimation enables qualitative and quantitative evaluation of the effectiveness of anti-aging products. Considering the parameters of subcutaneous layers, the proposed framework provides a reliable tool for TCA-peel therapy assessment; however, it can be successfully applied to other skin-condition-related problems. In this randomized controlled clinical trial, forty-six postmenopausal women were randomly assigned to the experimental and control groups. Women were treated four times at one-week intervals and applied skin cream daily between visits. The three month follow-up study enables measurement of the long-term effect of the therapy. According to the results, the TCA-based therapy increased epidermal (entry echo layer) thickness, indicating that the thinning process has slowed down and the skin’s condition has improved. An interesting outcome is the obtained growth in the intensity of the upper dermis in the experimental group, which might suggest a reduced photo-aging effect of TCA-peel and increased water content. The same conclusions connected with the anti-aging effect of TCA-peel can be drawn by observing the parameters describing the contribution of low and medium-intensity pixels in the upper dermis. The decreased share of low-intensity pixels and increased share of medium-intensity pixels in the upper dermis suggest a significant increase in local protein synthesis.

## Introduction

The last decades have brought new application areas for ultrasound imaging, including dermatology, allergology, cosmetology, aesthetic medicine, dermatological oncology, and rheumatology. In response to the appearing works in this area, the European Federation of Societies for Ultrasound in Medicine and Biology (EFSUMB) provides the Position Statement on Dermatologic Ultrasound^1^. According to this recommendation, the high- (> 15 MHz), very high- (> 20 MHz), and ultrahigh-frequency (30–70 MHz) transducers with enough spatial resolution can be introduced to study skin abnormalities. At the expense of a lower penetration depth, they improve the spatial resolution of the acquired images^2^, enabling the visualization of superficial structures and skin appendages. Therefore, high-frequency ultrasound (HFUS) opens up new opportunities for accurately examining skin and monitoring its treatment. It is applicable in skin tumor and inflammatory skin disease diagnosis assessment and staging, as well as the support for aesthetic procedures, which are mainly performed blindly.

According to the newest reports^3–7^ HFUS imaging is increasingly used in medical diagnosis, especially in dermatological oncology, which is the primary dermatological use of it. The assessment of skin cancer includes pre-operative diagnosis and early detection of neoplasms^5^. As claimed by the authors of^4,8,9^, it provides essential and objective information regarding tumor depth, lateral extension, and vascularity degree. The authors^4,5^ also mention the characteristic features of basal cell carcinoma (BCC) manifested in HFUS and the correlation of melanoma shape to its histologic subtype. Vergilio et al.^6^ described possible usage of HFUS in the diagnosis of cutaneous lymphomas, bullous pilomatrixoma, extramammary Paget disease, Bowen’s disease, atretic cephalocele, and infantile hemangiomas. The summary of possibly extracted tumor features in HFUS is given in^10^. The combined use of ultrasound and UCAs as a non-invasive, targeted, and safe method for delivering therapeutic drugs into melanoma is discussed in^7^.

The second widely explored area of HFUS application is the assessment of inflammatory skin diseases^3,5,11^. The increase in collagen deposition enables differentiating between the inflammatory and sclerotic phases in scleroderma. Analyzing the thickness of the subepidermal low echoic band (SLEB) in HFUS enables monitoring the treatment effects in psoriasis and atopic dermatitis (AD). In AD, the SLEB thickness correlates with the histologic degree of epidermal hyperkeratosis, parakeratosis, spongiosis, and intensity of inflammatory infiltrates as well as the provider-assessed EASI (eczema area and severity index) scores^5,11^. Ultrasound imaging is also used to diagnose hidradenitis suppurativa, enabling the detection of many lesions missed in palpation.

In aesthetic medicine, the HFUS is applicable to support the fillers injection, measure dermal thickness before the aesthetic procedure, or monitor the treatment results^6,12^. It is used as the control in anti-cellulite therapy and scar formation^6^. As reported in^12^, hyaluronic acid (HA), one of the typical fillers, is the most homogeneous and easily identified as a rounded anechoic space in HFUS. The HFUS enables the detection of nodules or granulomas and the introduction of hyaluronidase into areas of deposits and incorrect injection of HA. The long-term follow-up of longevity and diffusion pattern of two hyaluronic acid fillers using high-frequency ultrasound is described in^13^. According to the obtained results from the HFUS image analysis, the hyaluronic acid fillers generated by different cross-linking technologies display differential diffusion patterns in skin tissues. A similar analysis is performed by Tedeschi et al.^14^, where the authors apply the HFUS to evaluate the long-term effects of microinjections of HA on SLEB echogenicity. In both the studies^13,14^ the skin thickness measurements are carried out on a single HFUS image and in a single place. The analysis is performed manually^13^ or using built-in gray intensity-based segmentation methods^14^.

In cosmetology, among others the HFUS is used for skin aging assessment. The sought features in this application area are: the arrangement of collagen and elastin bundles, water loss, and presence/parameters of SLEB, which tend to change with age^6^. According to^15,16^ they are also sensitive to the botulinum toxin injections or topical vitamin C therapy utilized in anti-aging procedures. Vergilio et al.^6,15,17^ widely explore the correlations of skin parameters with age and sex. In^15^, the HFUS is evaluated in terms of the possibility of assessing the effectiveness of anti-aging products and procedures. The authors claim that HFUS is a crucial evaluative alternative for dermatological studies and the effectiveness of anti-aging products and treatments. The assessment of facial skin thickness associated with gender, age, and BMI in healthy adults using ultrasound is described in^18^. In^16^, the authors utilize the HFUS to evaluate the cutaneous changes induced by the topical use of a vitamin C complex—cream at the facial level. They use a Dermascan device (Cortex Technologies, Denmark) with a 20 MHz transducer. Apart from the thickness measurements (epidermal and dermal), there is a range of parameters described as beneficial for the skin assessment described in^16^: the number of low (LEP), medium (MEP), high echogenic pixels (HEP), and the ratio of the number of LEP in the upper dermis and lower dermis (LEPs/LEPi). Apart from^13,14^, the authors perform the thickness measurements three times—at three different sites of each image, making the analysis more reliable. The newest work in this area^19^ targets the in vivo evaluation of topical ascorbic acid application on skin aging by 50 MHz ultrasound (DUB SKinScanner75, Germany). The parameters considered are total skin, dermal and epidermal echogenicity, skin layer thickness, and surface roughness.

Since, despite all the benefits of using ultrasound, it is not commonly used in cosmetological practice, the number of papers describing its application in this area still needs to be increased. Therefore, this work presents the first usage of HFUS in assessing topical anti-aging therapy with trichloroacetic acid (TCA) chemical peel. Chemical peels are applied in anti-aging therapy to smooth the surface and remove the top layer of the callous epidermis. They are claimed^20,21^ to help eliminate pigmentary disorders, scars, and wrinkles and reduce the signs of chrono- and photo-aging. The methods for evaluating the efficacy of TCA in treating photo-aging are summarized in^21^. The degree of improvement is measured using: a quartile scale graded by dermatologists, global aesthetic improvement scale (GAIS), epidermal skin elasticity, hydration, melanin and erythema index, depth and volume of wrinkles, or lesion count.

The current work proposes quantitative HFUS parameters for evaluating the applied therapy. The measurements of HFUS images acquired with DUB SkinScanner75 (24 MHz transducer) include epidermal thickness and echogenicity, dermal echogenicity in different dermis regions - upper and lower dermis, LEP, MEP, and HEP values of different skin areas, as well as textural features describing the visual appearance of them. Utilizing the benefits of currently developed image processing techniques, the image selection and epidermis segmentation steps presented in our work are fully automated, making the analysis repeatable and reliable. In both steps, we introduce deep neural network models pre-trained on the publicly available HFUS image data^22^. The analysis aims to assess the usefulness of TCA in anti-aging therapy, but the universal character of the tool allows it to be used in other cosmetic applications.

## Materials and methods

All the experiments were performed following the protocol approved by the Bioethics Committee at the Academy of Physical Education in Katowice, Poland, under the reference number 3/2020 on 17 December 2020, and according to the ethical standards of the Declaration of Helsinki 2013. We confirmed that they were carried out in accordance with relevant guidelines and regulations. Informed consent was obtained from all subjects involved in the study. The trial is registered under the number: ISRCTN41899475; (doi.org10.1186/ISRCTN41899475). The HFUS imaging was performed by medical engineer with 5 years’ experience in HFUS ultrasound, graduated two dermatological HFUS courses. To reduce the possible influence of US operator and make the analysis repeatable, the US scanning was performed by the same person.

### Materials

The female facial skin image data were collected during a prospective, randomized, controlled, single-center clinical trial designed to compare the skin condition before and after TCA intervention in two parallel groups of women receiving TCA chemical peeling or placebo. The procedure was applied to women at the age of 59–65, whose eligibility to participate was assessed by the aesthetic medicine doctor against the following criteria: Fitzpatrick skin type II–III, no contraindications for chemical exfoliation, no previous treatments of TCA chemical peels and no previous exfoliating and rejuvenating treatments within six months. The inclusion and exclusion criteria are summarized in Table 1.

**Table 1:** Participants inclusion and exclusion criteria.

Inclusion	Exclusion
Age 59-65 year,	Contraindications for chemical exfoliation,
Fitzpatrick skin type II-III,	Malignancy,
No contraindications for chemical exfoliation (bacterial, viral or fungal infections, other inflammatory dermatoses such as psoriasis and topical dermatitis, topical retinoids treatment, keloid scarring tendency),	Pregnancy,
	Breastfeeding,
	Untreated hyperthyroidism and hypothyroidism,
	History of mental illness and unrealistic expectations about the results of the study,
No previous treatments of TCA chemical peels and no previous exfoliating and rejuvenating treatments within 6 months (including other chemical peels, ablative fractional laser, microneedling radiofrequency and other microneedling therapy etc.)	Inability to participate in a follow-up visits

**Figure 1:**
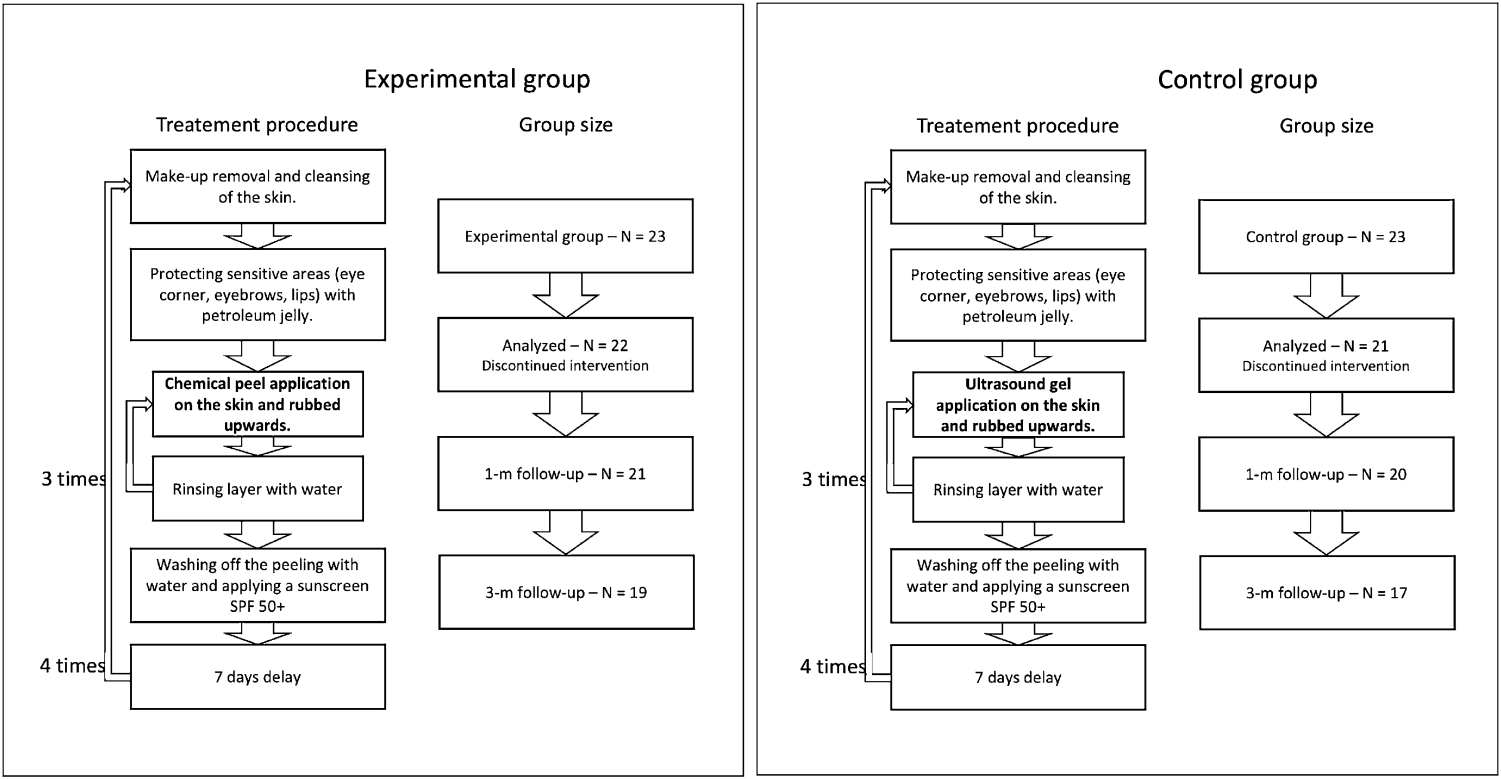
Summary of patient groups and treatment steps.

To blind the peel procedure, the personnel performing the procedure, data collectors, and data analysts did not know what substance was used, and the application procedure was the same in both the considered groups (per the TCA manufacturer recommendations). It includes make-up removal and cleansing of the skin, protecting sensitive areas with petroleum jelly, and applying the proper substances. Three layers of TCA (in the experimental group) and ultrasound gel (in the control group) were applied, with water rinsing in-between. After the last layer, the peeling/gel was washed off with water, and a cream with sunscreen SPF 50+ was applied. The application was carried out in four treatments every seven days. All the participants used soothing and moisturizing cream with SPF 50+ in daily care and up to three months after therapy. The novel TCA chemical peel (PQ Age Evolution®, Promoitalia, Italy) consists of: (1) Trichloroacetic acid $34\%$ (TCA), which helps to remove discoloration and small scars, smooth the skin, reduces blackheads and affects the synthesis of collagen, (2) Kojic acid $10\%$ with antibacterial and antifungal properties and combats discoloration, (3) $5\%$ urea peroxide with lightning properties, (4) Coenzyme Q10 $5\%$ with antioxidant properties.

The measurements were performed four times: at baseline—before the intervention, immediately after the last intervention, and one and three months post the last intervention. Patient groups with corresponding treatment procedures are summarized in Figure 1.

The required number of participants was evaluated based on the results of a pilot study, where five women were administered acid and five placebo. The assumptions for 2-way RM-ANOVA were met. Therefore the sample size was estimated using an eta-squared of the effect of hydration (measured using Multi Skin Test Center® MC750 B2, with the Corneometer® CM825 probe) for the between-factor analysis (eta-squared = 0.18). Setting alpha = 0.05, test power = 0.9, number of measurements = 6, number of groups = 2, correlation among repeated measures = 0.53, and eta-squared = 0.15, the total number of participants was 40 (at least 20 people in each group). Due to possible unpredictable and unavoidable factors, potentially reducing the group size, 23 people were qualified for each group, making the total sample size equal to 46. During the experiments, three participants were excluded from the analysis due to the absence or sunbathing, which is treated as possibly influencing the results. Consequently, the analysis was performed on 43 participants: 22 in the experimental group and 21 in the placebo group. The sample size agrees with the sizes described in the literature in similar experiments^13,14,16,19^.

**Figure 2:**
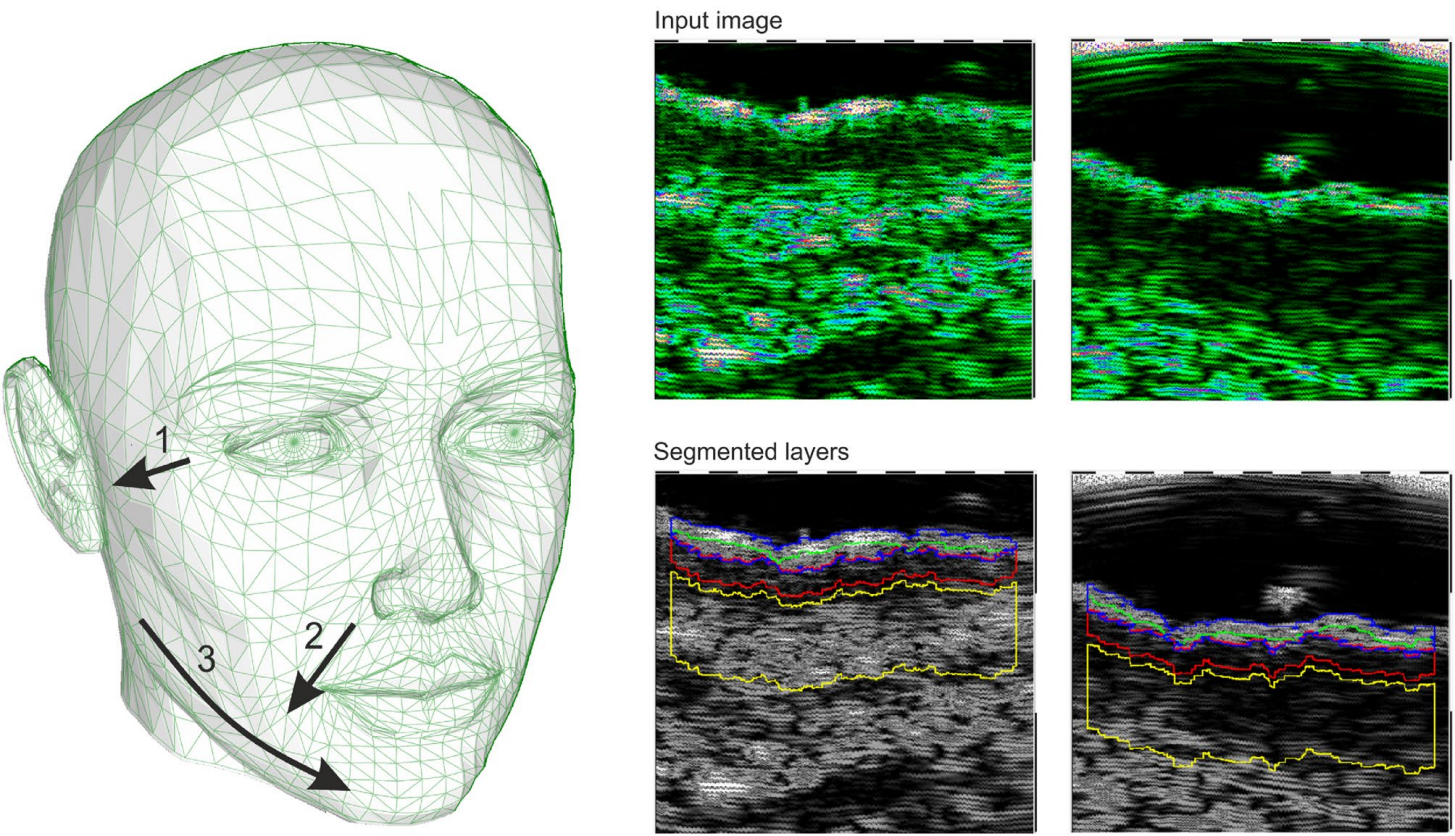
Left: facial model with superimposed image acquisition areas: the arrows indicate HFUS probe movement; Right: exemplary HFUS skin areas with segmented ROI for parameters estimation: blue - entry echo layer, green − 0.1 [mm] of upper epidermis, red − 0.2 [mm] ROI below the entry echo layer.

The HFUS images were acquired using DUB SkinScanner75 (tpm, taberna pro medicum GmbH, Germany) with a 24 MHz (B-mode frequency, 8 mm depth, and acoustic intensity level 40 dB) transducer in three locations on the patient’s face. The locations and ultrasound probe movement directions are visualized in Figure 2 by three arrows superimposed into a facial model. The image acquisition starts where the arrow begins and ends with the arrow end. Several dozen HFUS images were collected for each location in a single series. The original image resolution was equal $1386\times 3466$ [pix], and the pixel size is equal to $0.0093\times 0.0023$ [mm/pix] (axial $\times$ lateral). In total, 17425 HFUS frames of facial skin were acquired (available here^23^), from which only 3900 were considered relevant for the analysis (see Section *Image selection*).

### Skin parameters in ultrasound

Recent works^12,13,15,16,24^ indicate the parameters which can be measured in ultrasound and which provide information regarding changes in collagen structure, elasticity, and ultrastructural skin features. Reviewing the papers, we selected the set of skin properties that could be helpful to assess the anti-aging therapy^16,19,25^. In the next section, we propose the new parameters resulting from the image analysis, which can be successfully applied in this field.

#### Thickness

Verigilio et al.^15^ mention the relative reduction in skin thickness caused by aging in the photo-exposed areas. They report that the thickness of the sun-exposed area tends to increase after age 60, whereas the thickness of the photo-protected area tends to decrease. Based on the forearm area, the observation does not show the correlation of thickness with chronological but only photo-aging. The authors^15,24,26^ agree that there are no significant changes, correlating with age, in the epidermis and dermis thickness in photo-protected areas. On the other hand, the influence of anti-aging therapy on the skin layer thickness is undertaken in^16,19,25^. In the 1-year experiment described in^25^, retinaldehyde treatment induced a significant increase in epidermal (entry echo layer) thickness of the temple, compared to the control group $(p<0.01)$. The same outcomes are described in^16^, where the thickness of the epidermis (entry echo layer) increased significantly in the group of young participants at 60 days of vitamin C-based therapy compared with 40 days and before it. Unfortunately, the experiments from^19^ with topical ascorbic acid application did not confirm this.

TCA is described in literature^21^ as reducing the stratum corneum and decreasing the dermis thickness. According to works reviewed in^27^, there is no possibility of varying the different epidermis layers while using the 24 MHz transducer. It is debatable whether the entry echo corresponds to the total epidermis thickness or is only related to the stratum corneum, which is important in this study. As for the dermis layer, the need for a manually annotated repository for an automated segmentation algorithm and difficulties in differentiating between the dermis and lower layer makes the measurements impossible. Unfortunately, this problem is not addressed in the literature.

Considering all the above, we decided to use the entry echo layer thickness as the first parameter analyzed in the TCA assessment.

#### Echogenicity

The echogenicity of consecutive visible layers is widely described in literature^16,19,27^ as a valuable parameter in skin diagnosis. Based on the principles of ultrasound imaging, we can assume that bright or hyperechoic regions represent an increase in tissue density, while dark or hypoechoic areas represent a decrease. As reported in^27^ low echogenic region below the entry echo layer refers to the papillary dermis, whereas the brighter region below it stands for the reticular layer. The difference is related to the tighter packing of thick parallel arranged bundles of collagen in deeper layers of the skin. Moreover, the echogenicity of the dermis is influenced by several factors, and any pathological conditions associated with fiber accumulation will increase it^27^. For instance, botulinum toxin injections increase subcutaneous echogenicity^5^. Similarly, enhancement of fiber damage, inflammation, or water content decreases echogenicity. In the case of inflammatory dermatoses, a subepidermal low echogenic band (SLEB) is characteristic. However, it is also visible in sun-exposed skin in elderly individuals. On the other hand, the echogenicity tends to increase with age corresponding to increase collagen production^5^. Changes in skin echogenicity are widely reported in anti-aging therapies^13,14,16,19^. The experiments described in^16^ demonstrated increased echogenicity in both the epidermis and dermis after 40 days and, to a greater extent, after 60 days of topical vitamin C therapy. Tedeschi et al.^14^ reported an increase in SLEB echogenicity after mesotherapy with hyaluronic acid, and Vergilio et al.^19^ described an increase in high echogenic pixels in the dermis, after 30 days of anti-aging therapy compared to the placebo group. On the other hand, a decrease of dermal echogenicity in long-term follow-up after hyaluronic acid injection is reported in^13^.

In^21^, the authors mention the improved quality of elastic and collagen fibers due to the promotion of collagen fiber synthesis and increased water and glycosaminoglycan content in the dermis as an outcome of TCA peeling. Based on this claim and considering the above observations, we analyze the skin echogenicity in various skin areas (see Fig. 2): entry echo layer, outer part of entry echo layer - top 0.1 [mm], upper dermis − 0.2 [mm] below the entry echo layer (in some patients it corresponds to SLEB), dermis − 0.4 [mm] below the previous region.

Moreover, we propose a different intensity-based parameter - the ratio of the upper dermis to the epidermis (entry echo) intensities.

#### LEP, MEP, HEP

The numbers of pixels in various ranges of intensities were first described for skin diagnosis assessment in^16^, for analyzing the role of vitamin C in skin aging. LEP stands for the number of low, MEP for medium, and HEP for high echogenic pixels in the considered skin layers. Additionally, the authors analyze the LEPs/LEPi, as the ratio between the number of echogenic pixels in the upper and lower dermis. Assuming the intensity range in grayscale as 0–255, the LEP corresponds to the intensities range 0–30, the MEP 50–150 interval, and HEP 200–255.

According to^16^, LEP quantifies the degree of cutaneous hydration, inflammatory processes, solar elastosis, and collagen degeneration, MEP and HEP quantify the structures of collagen, elastin fibers, and microfibrils, and the LEPs/LEPi ratio allows an appreciation of the density and integrity of the extracellular matrix. The authors claim that the LEP decreased significantly in all age categories during and after the therapy, whereas both MEP and HEP increased. They explain this phenomenon by the significant increase in local protein synthesis. The LEPs/LEPi ratio does not vary significantly, increasing at the beginning and decreasing at the end of the considered time interval. In the placebo group, the LEP of the dermis increased. In contrast, the MEP and HEP decreased, which the authors explain as the effect of optimal hydration caused by applying the moisturizing cream.

Since the TCA peeling is described as^21^ stimulating fibroblasts to produce elastin, activate the skin stress response system and improve skin quality (better brightness, elasticity, and turgor), the bunch of above mention parameters are also calculated in our work.

#### Roughness

The superficial roughness as the parameter for assessing topical ascorbic acid application results is proposed in^19^. It measures the interfacial perimeter of the skin, which can be connected with wrinkling of its surface. According to^19^, the superficial roughness parameter presented a considerable decrease 30 days after the application compared to two hours after it.

One of the declared TCA effects is wrinkles reduction. As reported in^28^ in the group of patients with the preferable results using the global aesthetic improvement scale (GAIS) after the TCA therapy, the participants indicated lower laxity and fewer wrinkles. Therefore, in our study, the surface roughness is measured as well.

### Image processing methods

**Figure 3:**
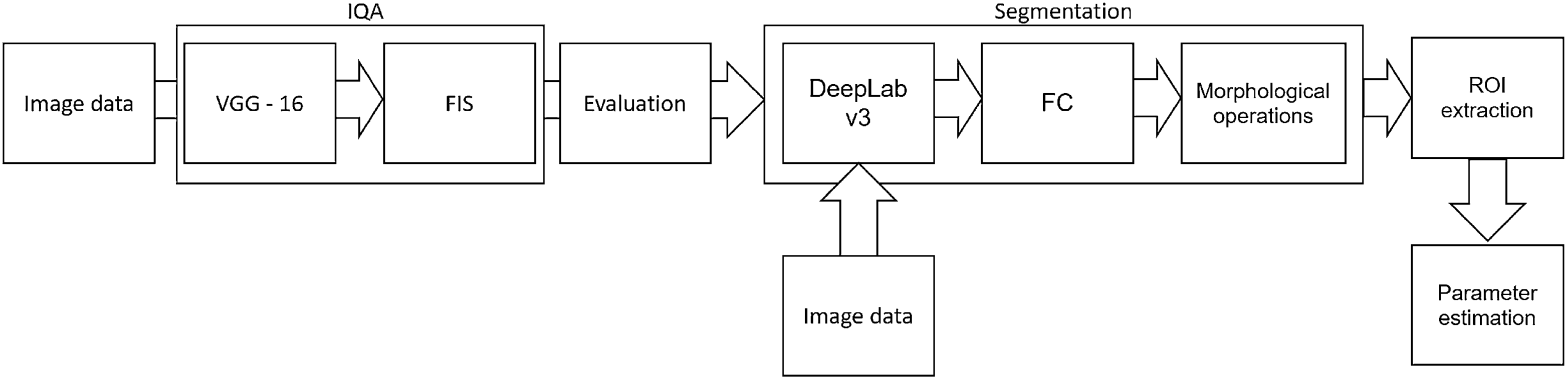
Block diagram of image processing method.

The above-referred methods^13,14,16,19^ describe the medical aspects of anti-aging therapies, utilizing the benefits of HFUS imaging. However, they lack reliable and repeatable methodology for image analysis. To meet this need, we propose a fully automated algorithm for quantitative assessment of the effectiveness of TCA peeling, which can be successfully applied in various applications connected with the evaluation of cutaneous conditions. The block diagram of the proposed algorithm is presented in Figure 3. The flow chart consists of two main parts: image quality assessment and segmentation steps. The segmented cutaneous areas are then utilized for skin parameters estimation in TCA therapy.

#### Image quality assessment

Since the collected image series includes both the image data suitable for further diagnosis or not, they were first evaluated using the deep learning-based image quality assessment algorithm^29^. The method utilizes the benefits of a fuzzy interference system (FIS) with the VGG-16 model. For further analysis, only the images classified by the system as ’perfectly ok’ with a probability over $90\%$ were considered. Since two experts also annotated the image data, the obtained classification results were compared with the exert decision. The proceeded HFUS data set was proven to consist of high-quality and diagnostically reliable image samples. The HFUS facial skin images used in this analysis are publicly available under^23^, and details concerning the data structure are described in^29^. Two examples of HFUS images are shown in Figure 2.

#### Segmentation

The skin parameters estimation is preceded by a fully automated segmentation step for reliable and repeatable analysis. Based on the previous work^30^, the developed method applies DeepLab v3+ network with ResNet-50 backbone followed by fuzzy connectedness step. The DeepLab v3+ network is trained using the HFUS images publicly available in^22^. As reported in^30^, for this data^22^, the segmentation accuracy (Dice index) of the entry echo layer was equal to 0.919. The training data were acquired with the same US machine (DUB SkinScanner75) but with different transducers frequency (75 MHz). Therefore, some additional post-processing steps are introduced. First, to avoid the influence of artifacts on the image border, the resulting masks are limited to the central part of the image - excluding 20 [pix] from each border area. It reduces the 12.8898 [mm] width image of 0.372 [mm] ($2\%$). Second, morphological operations are used to remove small objects. The exemplary segmentation results are delineated in blue in Figure 2. Before further analysis, the expert in HFUS skin imaging visually analyzes the segmented regions.

#### Skin parameters estimation

**Figure 4:**
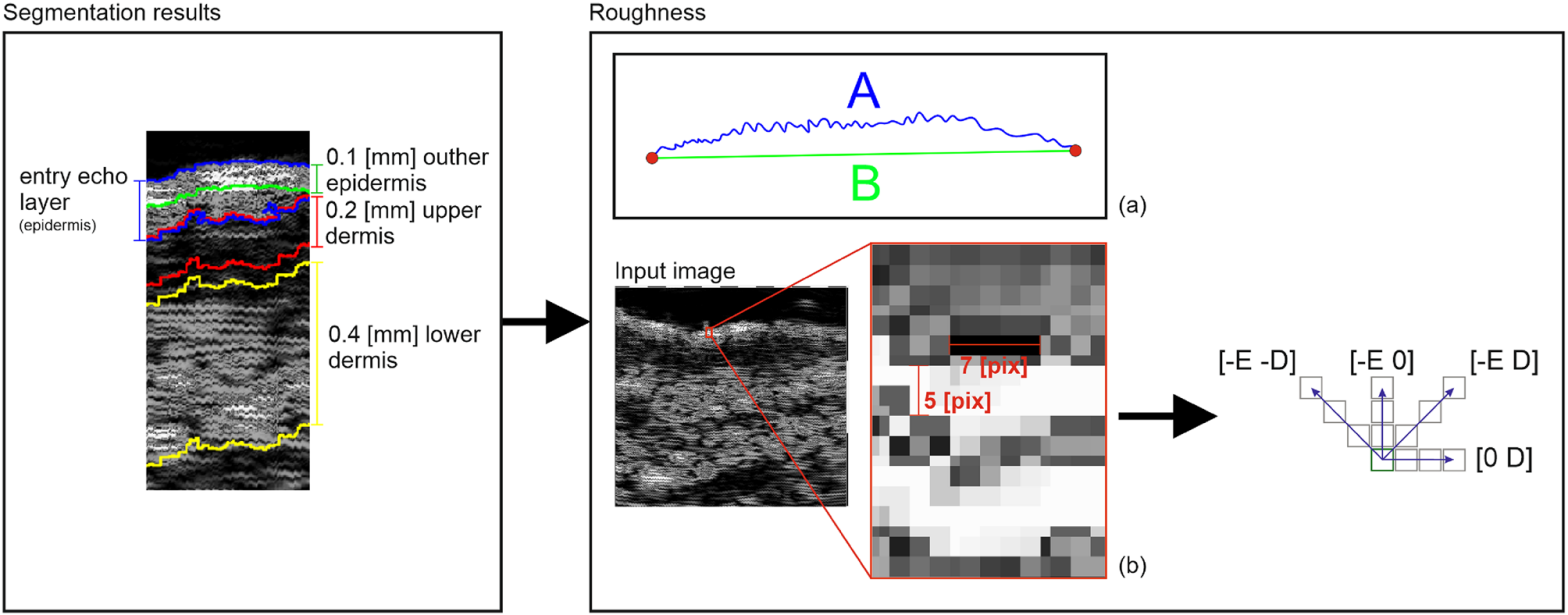
Roughness parameters estimation.

The segmentation mentioned above results in the entry echo area, and the other cutaneous regions are defined with respect to it. Since all the considered skin images are registered using the same acquisition parameters and all the HFUS images contain the visible ruler graduated in millimeters, direct comparison of echogenicity levels and estimation of actual pixel size is possible. Assuming that the skin layers are almost parallel, the regions for the echogenicity are defined considering the segmented entry echo layer and the previously estimated pixel size at different depth levels visualized in Figures 2 and 4. Because the automated segmentation step is limited to the entry echo area, the changes in thickness are evaluated only for this region.

Since, in this analysis stage, we cannot assume the constant thickness of the consecutive layers (we even expect some changes), the absolute number of pixels used for LEP, MEP, and HEP values estimation can lead to gross errors. Therefore, in our approach, all these parameters are normalized by the number of pixels creating the analyzed areas. They are estimated for all the skin layers visualized in Figure 2. The exemplary nLEP for the entry echo layer is defined as:

1 $$\begin{aligned} nLEP = \frac{n_{30epi}}{n_{epi}} * 100\%, \end{aligned}$$

where:

$n_{30epi}$—number of pixels belonging to the entry echo layer with intensity level in [0–30], $n_{epi}$—number of pixels belonging to the entry echo layer.

According to^19^, surface roughness is considered a valuable measure for the anti-aging therapy assessment. Therefore, we propose two different roughness definitions relating to various aspects of skin analysis. The first one, more intuitive, is the ratio of the length of the actual edge of the segmented entry echo layer to the width of this layer ($\frac{A}{B}$ in Fig. 4a). It relates directly to the roughness of the skin surface, whereas the second definition refers to the skin’s internal structure. It measures the roughness based on the texture parameters and gray level co-occurrence matrix (GLCM)^31^. The GLCM includes the information on how often a pixel with the intensity *a* value occurs in a predefined spatial relationship to a pixel with the value *b* (see Figure 4). Based on numerical experiments and considering the characteristic pattern appearing in the segmented layers, the GLCM parameters *E* and *D* visualized in Figure 4 are set to 5 and 7, respectively, and the number of utilized gray levels is limited to 128. Based on the obtained GLCM parameters, the following texture measures are analyzed: contrast, representing the local variations in the GLCM; correlation, measuring joint probability occurrence of the specified pixel pairs; energy, describing the uniformity of the analyzed region; and homogeneity. Additionally, entropy, which measures the disorder within the analyzed region, is considered.

### Statistical analysis

**Figure 5:**
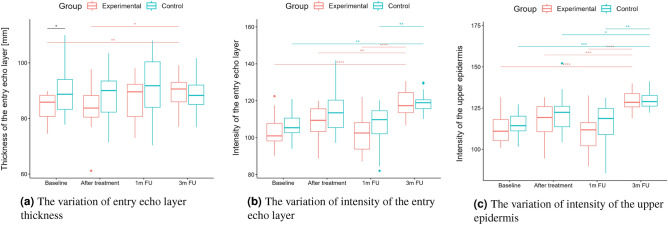
Thickness and intensities.

**Figure 6:**
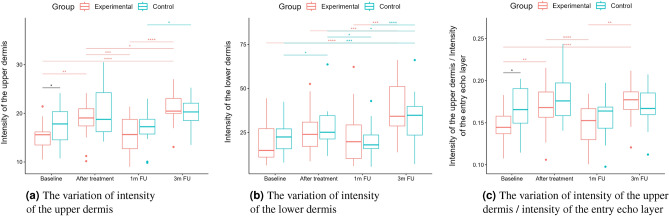
Intensities.

**Figure 7:**
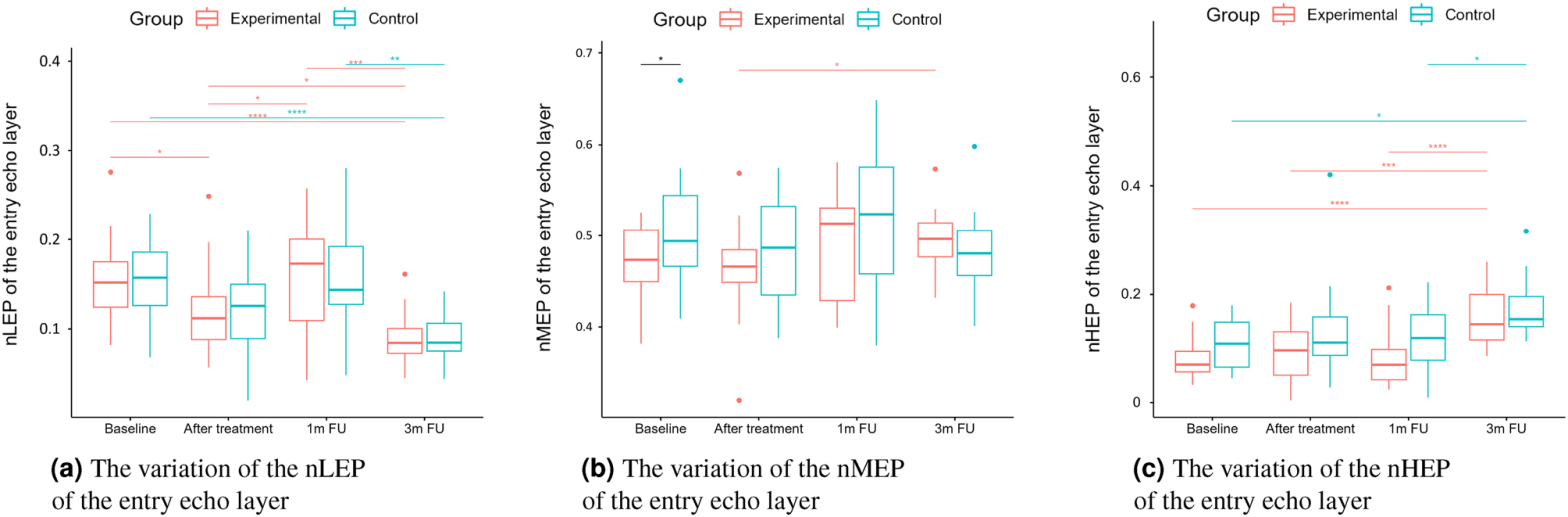
nLEP, nMEP, nHEP (entry echo layer).

Statistical analysis was performed based on the results obtained from 43 women (22 in the experimental and 21 in the control group) in a double-blinded study. The skin hydration and demographic features of women in these two groups revealed that at baseline, the groups were not statistically significantly different for any of the considered variables ($p>0.05$ in all cases). However, some of the cutaneous parameters resulted from the HFUS analysis differed significantly from the beginning of the experiments (see Fig. 6a,c,7b,5a,11a–c,12b).

**Figure 8:**
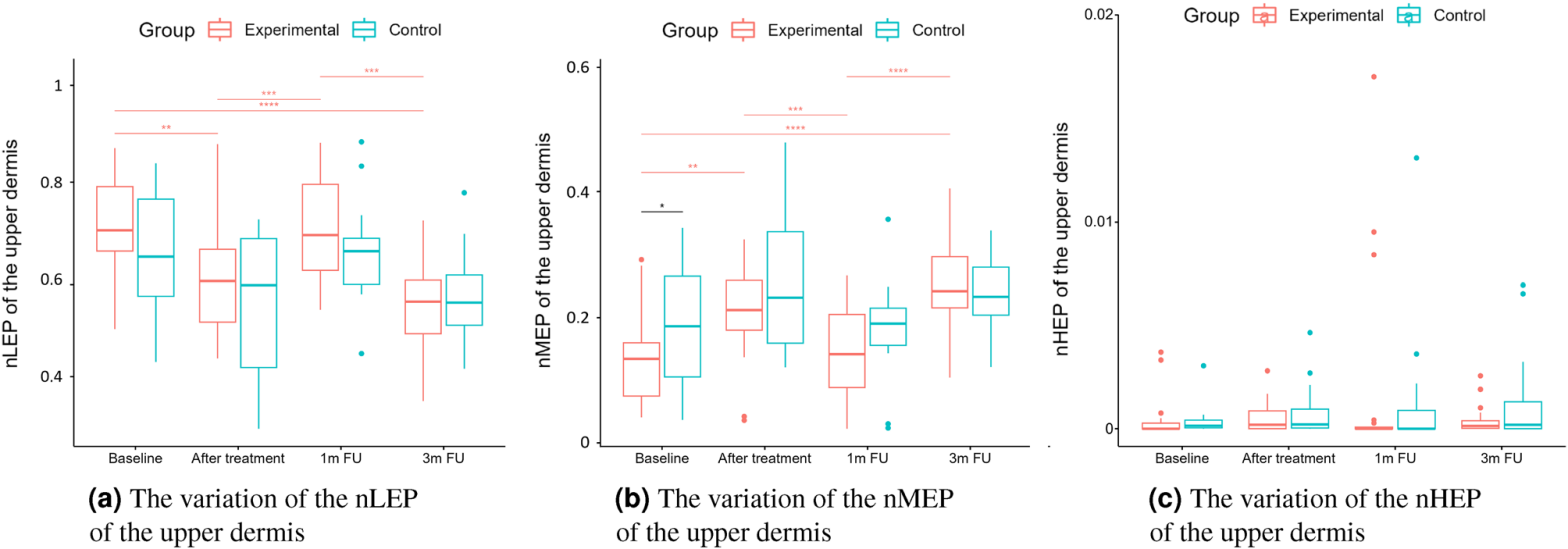
nLEP, nMEP, nHEP (upper dermis).

**Figure 9:**
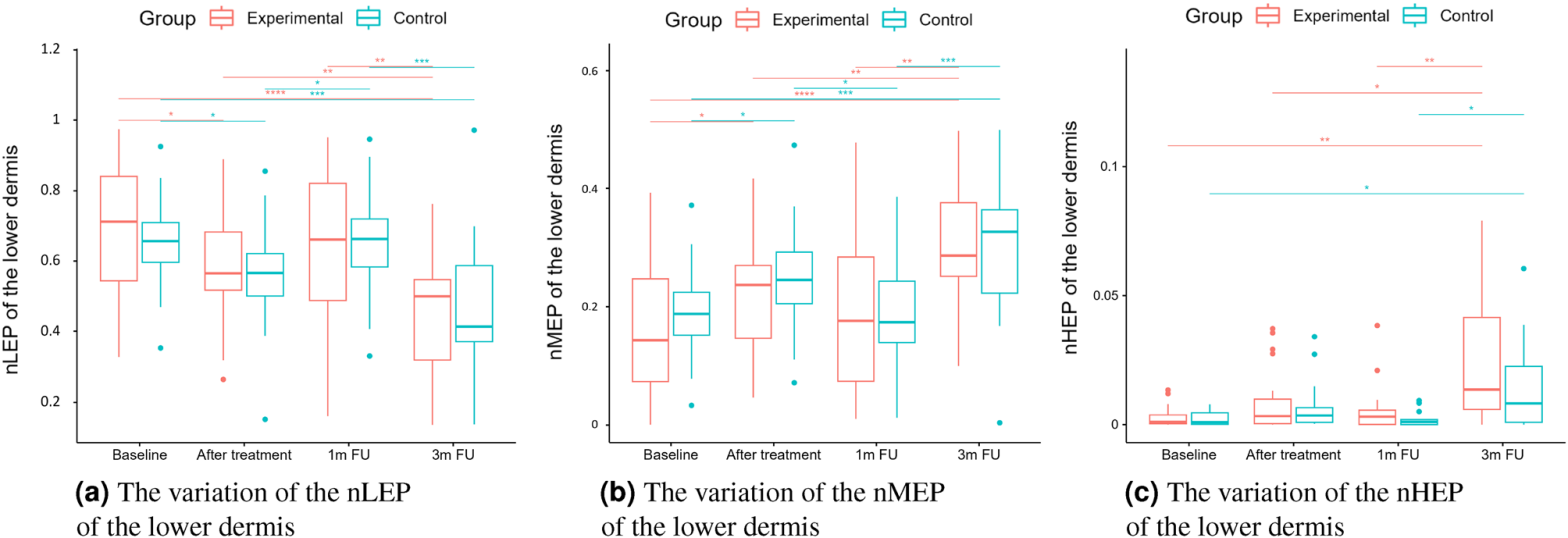
nLEP, nMEP, nHEP (lower dermis).

**Figure 10:**
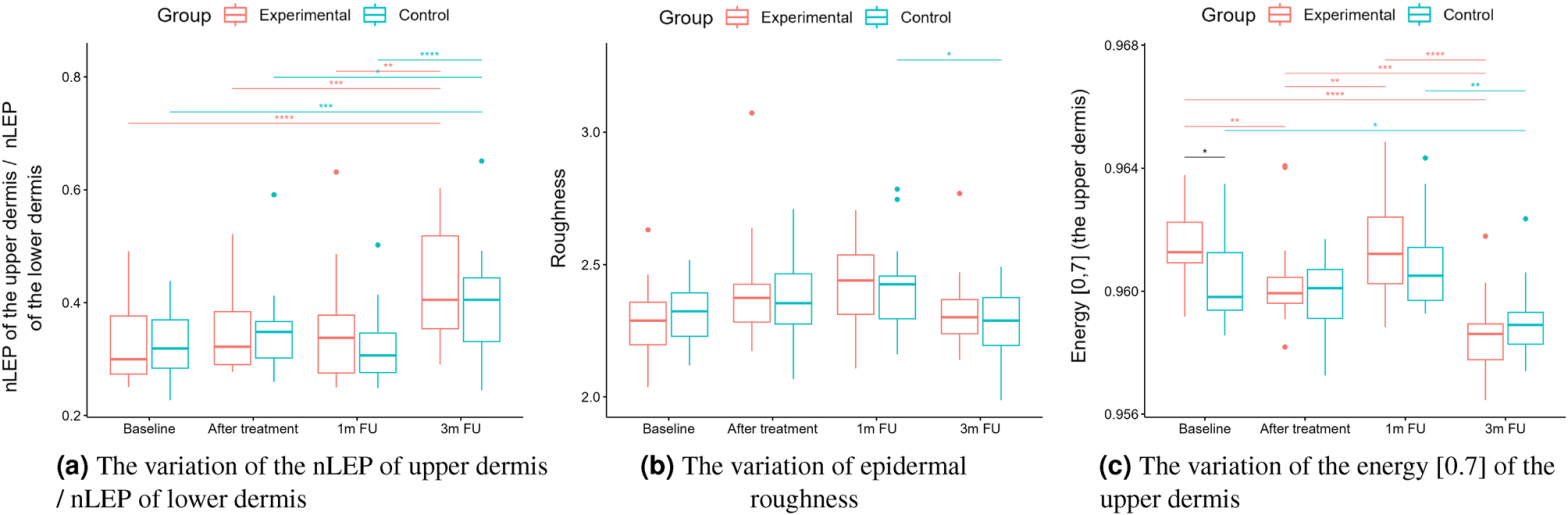
nLEP, nMEP, nHEP ratio, roughness, energy.

A two-way repeated measure ANOVA was performed to evaluate the effect of TCA peel administration on HFUS skin parameters measured with DUBSkinScanner75 at four-time points compared to the control group. The obtained results are shown in Figures 5, 6, 7, 8, 9, 10.

In the case of confirmed interaction, the differences between groups were assessed separately at individual time points. No interaction indicates that the differences between the group means did not change significantly over time - did not depend on the measurement time point. Each statistically significant effect was assessed in terms of its size, and minor effects, being clinically irrelevant, were not reported in this paper.

Boxplots present the median, the first and third quartile, and the whiskers indicate the outliers range. Statistically significant differences are indicated with stars (*p<0.05; **p<0.01; ***p<0.001; ****p<0.0001), marked red for the experimental group, cyan for the control group, and black for between-groups comparisons.

**Figure 11:**
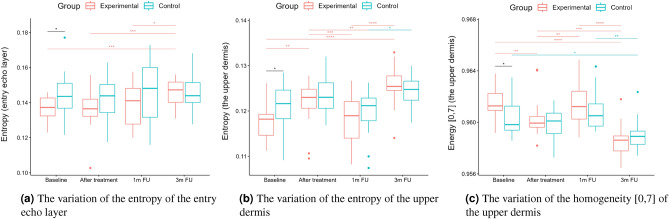
Entropy, homogeneity.

**Figure 12:**
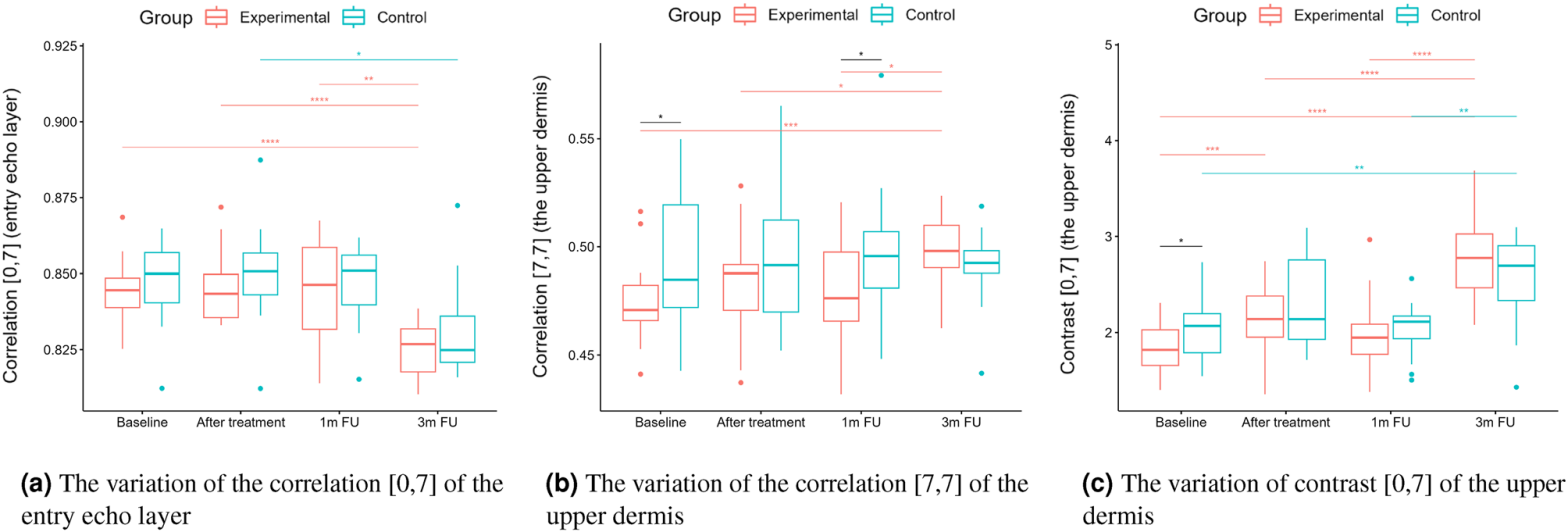
Correlation, contrast.

### Bioethical approval & trial registration

The study was approved by the Research Bioethics Committee of the Academy of Physical Education in Katowice (Mikolowska 72a Street, 40-065 Katowice, Poland) under the reference number 3/2020 on 17 December 2020. Informed consent was obtained from all subjects involved in the study. The trial is registered under the number: ISRCTN41899475; (10.1186/ISRCTN41899475).

## Results

According to the statistical analysis results, the groups differed statistically significantly at baseline regarding entry echo layer thickness (Fig. 5a), which was lower in the experimental group. However, only in the experimental group the thickness value is statistically higher when comparing baseline and three months follow-up.

As for the intensity (Figs. 5b and 6b), the groups did not differ statistically significantly at most of the time points (one exception is the upper dermis at baseline). In both the considered groups, the intensities of the entry echo layer (Fig. 5b,c) and lower dermis (Fig. 6b) increased statistically significant. However, only for the upper dermis (Fig. 6a), the intensity increased statistically significantly in the experimental group and remained almost constant in the control group. The same effect can be observed in the upper dermis’s intensity normalized to the epidermal layer’s intensity (Fig. 6c).

As we can notice in the case of the entry echo layer (Fig. 7), the nLEP value decreased statistically significantly in both the experimental and control group (comparing the baseline and three months follow-up), the nHEP increased in both groups but stronger in the experimental group and the nMEP remained almost at the same level. For the upper dermis (Fig. 8), changes in nLEP and nMEP are statistically significant only for the experimental group. The nLEP value (Fig. 8a) decreased directly after treatment, then increased in one-month follow-up to decrease again in three months follow-up. The opposite effect can be observed in nMEP (Fig. 8b). In the case of the lower dermis, there are no statistically significant differences between groups in nLEP, nMEP, and HEP parameters. Comparing the baseline and three months post-intervention, the nLEP decreased, whereas nMEP and nHEP increased.

There are no statistically significant differences between the groups and only small within the groups in terms of epidermal roughness (Fig. 10b, calculated according to Fig. 4 (a)), which increased (but not statistically significant) after the treatment and decreased in three months follow up in both experimental and control group.

From the set of measured texture parameters, we selected only a few examples that represent the general trend (Figs. 11a and 12c). The values in ’[,]’ in Figs. 11 and  12 refer to the GLCM parameters visualized in Figure 4. The groups differed statistically significantly at the baseline regarding entropy, homogeneity, and correlation. However, only the experimental group displayed significantly increased entropy when comparing the baseline and three months follow-up of the entry echo layer and upper dermis (Fig. 11a,b). The homogeneity of the upper dermis (Fig. 11c) tends to decrease in both the groups, however stronger in the experimental one. The correlation [0,7] measured for the entry echo layer displays (Fig. 12a) a decrease in both the groups, but it is statistically significant only in the experimental group. Similar results were obtained for the upper dermis. An inverse effect for all the considered layers can be observed for the correlation [7,7], an example for the upper dermis shown in Figure 12b. Again, it significantly increased only in the experimental group. The contrast of the upper dermis (Fig. 12c) increased in both groups but stronger in the experimental group. The same overall texture parameter change over time was observed for the lower dermis (the entropy, correlation [7,7] and contrast [0,7] increased in both groups, and the homogeneity and correlation [0,7] decreased). However, the changes were at the same level for both groups and the differences between groups were not statistically significant.

## Discussion

The effectiveness of the TCA-based peels is confirmed by the results of a recent review of clinical trials by Sithoang et al.^21^. The TCA-based efficacy in treating photoaging was evaluated with different measuring tools, including a cutometer, corneometer, mexameter, etc., and visual assessment - global aesthetic improvement scale (GAIS).

In our work, as the first, we propose a fully automated method for TCA-based peel assessment using HFUS. The HFUS is proven helpful in dermatology and cosmetology, and it provides crucial information in the evaluation of anti-aging products and procedures^16,19^. However, the quantitative analysis of the HFUS data requires a reliable image processing algorithm to analyze multiple images acquired for a single patient. To meet this need, the developed tool combines IQA assessment algorithm with the image segmentation method enabling quantitative and qualitative analysis of cutaneous tissue in TCA-based therapy. The analysis of subepidermal layers complements the classical methods described in^21^. Moreover, the universal character of the proposed processing flowchart makes it possible to apply it in various skin-related therapy assessments.

Since the image quality assessment results are verified in our previous work^29^ and the adopted segmentation method^30^ resulted in Dice Index equal to 0.919 for the original data, and the expert accepts the currently segmented areas, the evaluation of their performance is not discussed in this paper. However, it is worth mentioning that after the image selection step, the automated entry echo layer segmentation and cutaneous parameter estimation was successfully performed for the set of 3900, enabling credible statistical analysis. On the other hand, the automated segmentation of the dermis remains an area for development.

According to the obtained results, the TCA-based therapy increased epidermal (entry echo layer) thickness. The same anti-aging effect is described in^16^ in topical vitamin-C therapy. Skin aging within the epidermis manifests in the thinning of its living layers, while the thickness of the stratum corneum increases in some places. Since the peeling removes the accumulated corneocytes and the stratum corneum, and the epidermis in the experimental group is thicker after the treatment, it may indicate that the thinning process has slowed down and the skin’s condition has improved. Due to the fact that the ultrasound does not allow the differentiation of epidermis layers, it can also be assumed that the increase in thickness is related to the exfoliation process. However, this is not confirmed by the “roughness” analysis, which support the first version of the conclusions. The novel TCA formula stimulates internal renewal and regenerates the deepest layers of the skin in a very gentle way without damaging the skin surface.

As mentioned before, the analysis of roughness (Fig. 10b) does not suggest any improvement in this aspect, and the slightly increasing value (not statistically significant) in both the groups after the therapy and return to the baseline level in three months follow up can probably be attributed to facial massage during application.

The intensities of the entry echo layer and lower dermis increased in both the considered groups, which can be attributed to the post-treatment recommendations (avoiding the sun, using sunscreen, stopping skin peeling, using mild cleansers, etc.). An interesting outcome of this study is the obtained growth in the intensity of the upper dermis, where the difference between baseline and three months follow-up are statistically significant only for the experimental group. The examined groups of patients manifest the presence (weaker or stronger) of subepidermal low echogenicity band layer in the upper dermis, being the result of photo-aging. Its increasing brightness in experimental group might suggest reduced photo-aging effect of TCA-peel and increased water content. According to^16^, the loss of the proteoglycans’ hydrophilic properties due to aging and glycation accumulates in the papillary dermis and forms the SLEB. The SLEB is, therefore, an ultrasonographic aging parameter quantifying local morphological changes (cutaneous laxity, elastosis). The same conclusions connected with the anti-aging effect of TCA-peel can be obtained by observing the nLEP and nMEP values of the upper dermis. After the therapy, the nLEP decreases significantly, whereas the nMEP increases, which suggests a significant increase in local protein synthesis.

However, standing alone, nLEP, nMEP, or nHEP values do not provide any information concerning the structure of the analyzed regions and may lead to erroneous conclusions. Therefore, only their analysis, supplemented by the texture parameters, assures reliable information for anti-aging product assessment. The increased entropy of the upper dermis connected with decreased homogeneity and energy, and increased contrast, followed by the significantly decreased nLEP and increased nMEP, suggest appearing isolated hyper-insensitive areas which are connected with the promotion of collagen fiber synthesis mentioned in^21^. On the other hand, a high level of correlation proves the existence of a linear relationship between the brightness of neighboring pixels - the image in a given direction brightens, darkens, or, in a particular case, does not change its brightness. A high correlation value (ca. 0.825 to 0.850) along the [0.7] direction may indicate the presence of longitudinally arranged structures. A decrease in this value, along with an increase in the correlation in other directions (e.g. [7,7]), especially for the upper dermis layer, combined with reduced homogeneity and higher entropy, suggest a change in the structure of this area from longitudinal bands to more numerous, but shorter bands with high intensity - the homogeneity of this area, therefore, increases, suggesting elastic fiber rearrangement.

To summarize the above, the fully automated HFUS image processing method enables repeatable analysis of a large portion of pre-selected, high-quality images, making the parameterization step reliable. The extended set of HFUS parameters makes it possible to conclude about the internal skin structure and can be easily applied to assess other topical therapies.

Some limitation of this approach is the lack of the lower layers segmentation step, which should be the successive extensions of this work. Moreover, the utility of working with various scanners should be added. However, it requires a range of annotated data and the same time to prepare them.

It is worth mentioning that according to the newest studies in skin ultrasound^32,33^, also US elastography may find an application in dermatology and aesthetic medicine. It enables direct evaluation of various pathological or natural processes, providing information on facial skin elasticity. However, the combination of US and elastography, most welcome in our approach, requires numerous annotated data for training the segmentation model.

## Data Availability

The HFUS image data annotated by the expert as well as the FIS and trained VGG models are publicly available in Mendeley Data, 10.17632/td8r3ty79b.1.
